# Absence of p21^(WAF1/CIP1/SDI1)^ protects against osteopenia and minimizes bone loss after ovariectomy in a mouse model

**DOI:** 10.1371/journal.pone.0215018

**Published:** 2019-04-10

**Authors:** Priyatha Premnath, Leah Ferrie, Dante Louie, Steven Boyd, Roman Krawetz

**Affiliations:** 1 McCaig Institute for Bone and Joint Health, Cumming School of Medicine, University of Calgary, Calgary, Alberta, Canada; 2 Department of Cell Biology and Anatomy, Cumming School of Medicine, University of Calgary, Calgary, Alberta, Canada; 3 Biomedical Engineering Graduate Program, University of Calgary, Calgary, Alberta, Canada; 4 Department of Radiology, Cumming School of Medicine, University of Calgary, Calgary, Alberta, Canada; 5 Department of Surgery, Cumming School of Medicine, University of Calgary, Calgary, Alberta, Canada; Universite de Nantes, FRANCE

## Abstract

p21^(WAF1/CIP1/SDI1)^ is a critical sentinel of the cell cycle that plays an important role in determining cell fate with respect to proliferation, differentiation and apoptosis. Recent studies have demonstrated that inhibition/loss of p21 promotes osteo-chondro differentiation in progenitor/stem cells, and that p21 knockout (p21^-/-^) mice demonstrate enhanced bone regeneration compared to wild-type controls after a non-critical size defect. It was therefore hypothesized that the absence of p21 may also protect against bone loss through enhancing bone formation, tilting the balance away from bone resorption, in an ovariectomy-induced osteopenia mouse model, investigated via microCT imaging. While p21^-/-^ mice demonstrated significantly less bone loss after ovariectomy compared to wild-type controls, no increase in the number osteoclasts or osteoblasts in the bone or bone marrow was observed, nor was there an increase in osteoclast activity. Therefore, while the absence of p21 protected mice against estrogen mediated bone loss, the mechanisms/pathways responsible remained elusive. This study demonstrates that p21 may play a significant role in bone remodeling, and a better understanding of how the p21 pathway regulates bone anabolism and catabolism could lead to novel therapies for osteoporosis in the future.

## Introduction

Osteoporosis is characterized by the deterioration of bone architecture and consequent loss in bone strength, and its onset is based on age and/or menopause. In menopause, a deficiency in estrogen exacerbates osteoclast resorption compared to matrix being laid down by osteoblasts by elevating cytokines such as tumor necrosis factor α(TNF-α), interleukin-1(IL-1), IL-6 and interferon γ (IFN-γ) [1]. Current therapies for osteoporosis directly target osteoclasts and impede resorption via pharmacological anti-resorptive drugs such as bisphosphonates. Other strategies include treatment with selective estrogen receptor modulators or receptor activator of nuclear factor kappa B ligand inhibitors, but all have considerable disadvantages [1,2]. The primary drawback with most current treatment options is that while they prevent further resorption of bone, they do not promote the regeneration of bone that has already been lost to the disease process, leaving the bone in a weakened state.

To stimulate bone formation, stem cell therapies, in particular mesenchymal stem cells (MSCs) provide an attractive option. MSCs are a specialized cell type found throughout the bone (bone marrow, fat, connective tissue) which have the ability to form new bone tissue *in vitro* and *in vivo*. In pre-clinical studies, osteoporotic animals injected with MSCs demonstrate an increase in bone mineral density post-implantation over time [3]. Osteoporosis however is a systemic disease and the implantation of MSCs may not always provide a localized response if the root cause behind the disease remains untreated. As a potential alternative, there has been an effort to instigate endogenous tissue resident stem cells and/or terminally differentiated cells (e.g. osteoblasts) to enhance bone forming/repair capacity *in vivo*. In this regard Liu et al. demonstrated that microRNA (miR)-106b negatively regulates osteogenic differentiation of MSCs by interacting with bone morphogenetic protein 2 (BMP2). Therefore, by silencing miR-106b they were able to enhance bone formation and reduce bone resorption [4]. In another study, miR-34a overexpression in osteoclasts was found to attenuate bone loss during osteopenia [5]. Similarly, several other pathways have been linked to bone healing and regeneration, including the p53 signaling pathway [6]. An important component of the p53 signaling pathway is p21, which binds to cyclin-dependent kinases and inhibits their activity thereby preventing phosphorylation of retinoblastoma protein (pRB), preventing expression of genes associated with cell proliferation [7]. Therefore, inhibition of p21, removes inhibition of pRB and leads to an increase in cell proliferation. Interestingly, inhibition of p21 has also been implicated in tissue repair/regeneration, including increased ear hole closure [8] and liver regeneration [9], yet, loss of p21 is also associated with delayed regeneration of skeletal muscle [10]. In specific regards to the musculoskeletal system, the absence of p21 in hypertrophic chondrocytes results in apoptosis during endochondral ossification, thereby suggesting that p21 also pays a role in skeletal development [11]. Supporting this hypothesis, recent work from our lab demonstrated that the absence of p21 led to enhanced bone regeneration after a fracture injury [12]. Taken together, these results strongly suggest that p21 plays a role in development and maintenance of the musculoskeletal system. However, it remains unknown if p21 plays a role in bone homeostasis and/or the loss of p21 could counteract the effects of the estrogen-induced bone loss. Therefore, the aim of this research study was to determine if mice lacking p21 expression were protected against the development of ovariectomy (OVX)-induced osteopenia in a mouse model.

## Materials and methods

### Animal model

All animal procedures were reviewed and approved by the University of Calgary Animal Care Committee. Any person in contact with mice underwent special mouse handling and surgery training prior to the experiment. p21^-/-^ mice (B6;129S2-Cdkn1atm1Tyj/J, Jackson labs) backcrossed onto a C57BL/6 background and wild-type C57BL/6 mice (Jackson labs) were employed in this study. Both mice strains were 8–12 weeks of age at the time of their first Micro-CT scan. For OVX surgery, mice were administered Buprenorphine (0.05 mg/kg) and the surgery was conducted while the mice were administered isoflurane. The mice were given 0.05mg/Kg of Buprenorphine 12 hours subsequent to the surgery for pain management. The mice were monitored for any adverse reactions. Mice were kept in a mouse housing colony where cages are monitored every day. They were given free access to clean water and food. The mice were monitored for any adverse reactions. Three mice in the study died due to causes unrelated to the experiment. Mice were sacrificed via cervical dislocation at the end of the experiment. Humane endpoints were used in our experiment if a mouse was found to be in distress/pain or showed signs of malocclusion (NC3Rs ARRIVE Guidelines Checklist).

### *In vivo* micro-computed tomography (microCT)

Our previous studies have demonstrated that the radiation dose per microCT scan is 360 mGy and that a total of 4 scans does not negatively affect bone [13]. In order to prevent exposure to excessive radiation the mice were divided into two groups: mice that underwent a Pre-OVX scan, 1, 4 and 8 week post-OVX scan, and mice that underwent 1, 4, 8 and 16 week post-OVX scans. Therefore, the total number of mice per strain per timepoint were as follows: Pre-OVX (p21^-/-^, n = 6; C57BL/6, n = 5), Week 1 (p21^-/-^, n = 9; C57BL/6, n = 8), Week 4 (p21^-/-^, n = 7; C57BL/6, n = 8), Week 8 (p21^-/-^, n = 7; C57BL/6, n = 8), Week 16 (p21^-/-^, n = 4; C57BL/6 n = 4). Mouse mortality resulted in the uneven numbers in mouse groups. Trabecular bone morphology and bone mineral density (BMD) was measured using the right tibia of the mice. For the scanning period (approximately 20 minutes) mice were anesthetized using 1.5 vol/vol% isoflurane with 1L/min oxygen. The proximal 6.36mm of the right limb and left limb was scanned at an isotropic resolution of 15μm (μCT 40, Scanco Medical AG, Basserdorf, Switzerland) at a tube voltage of 45 kVp, tube current of 133 μA, integration time of 200ms, consisting of 1000 projections over 180° and reconstructed on a 2048 X 2048 matrix. The scanner was calibrated using hydroxyapatite phantoms. After the scans were completed, cortical and trabecular bone regions were extracted using Image Processing Language (IPL V5.08b, Scanco Medical, Brüttisellen, Switzerland) via segmentation [12]. The mean gray-scale values of voxels in the cortical and trabecular region were converted to HA/cm^3^ to be reported as bone mineral density. Trabecular bone volume ratio (BV/TV), trabecular thickness (Tb.Th), trabecular separation (Tb.Sp), trabecular number (Tb.N), structure model index (SMI) and trabecular connectivity density (Conn.D) were calculated after segmentation.

### Flow cytometry

The number of MSCs, osteoclasts and osteoblasts were enumerated via flow cytometry. Immediately after the mice were euthanized, the left tibiae were dissected and the bone marrow was flushed with a 26-gauge needle. The resulting cell suspension was treated with red blood cell lysis buffer to remove red blood cells (1X ammonium chloride solution). The remaining cells were stained with Sca1 (BD Biosciences) and CD140a (BD Biosciences) (MSCs markers), or CD115 (BD Biosciences), CD53 (BD Biosciences) and RANKL/TRANCE (BD Biosciences). Cells positive for RANKL and negative for CD53 were considered osteoblasts [14], and cells positive for CD115 and negative for CD53 were considered osteoclasts [15].

### Osteoclast resorption assay

In order to functionally assess osteoclasts, a Corning osteo assay^®^ was employed (Corning). Mice femurs were flushed 8 weeks post OVX, and all the cells obtained from the flush were plated onto an osteo plate following manufacturer’s instructions. M-CSF (macrophage colony stimulating factor) (30ng/ml) and RANKL (50ng/ml) was added to the media, and the media was replenished every 2 days until 7 days. Following this, the media was aspirated and plates were treated with 10% bleach solution. The wells were washed thrice with water and treated with 1% toluidine solution to visualize pits. The area of the pits was measured via an automated process created on Image J (1.49, NIH, USA).

### Histology and immunohistochemistry

Mice were sacrificed at 4 and 8 weeks and their right tibiae were dissected out. The specimens were fixed in formalin for 7 days followed by decalcification in Cal-Ex™ (Fisher Scientific) for 10 days where the solution was changed every alternate day. Samples processed for immunofluorescence were decalcified in 10% EDTA solution for two weeks. This was followed by tissue processing for paraffin sectioning. Samples were divided equally to be embedded in a sagittal as well as in a transverse fashion. Ten micron sections were cut using a microtome (Leica) and mounted on Superfrost Plus slides (Fisher). Slides were de-paraffinized and stained with saffarin-O and fast green (Electron microscopy sciences) to visualize proteoglycans and bone.

For immunofluorescence, slides were de-paraffinized in CitriSolv (Fisher Scientific) then rehydrated through graded ethanol to water. This was followed by antigen retrieval with 10mM sodium citrate and blocking with 100μl goat serum:50mL TRIS-buffered saline, 0.1% Tween 20 (TBST) for one hour. After washes with TBST, primary antibody, TRAP (Tartrate-resistant acid phosphate) (ProSci Incorporated) or BSP (Bone sialoprotein) (Developmental Studies Hybridoma Bank) was applied to sections and left overnight in a dehumidifier at 4°C. In case of staining for Ki67 (Ki-67 Monoclonal Antibody (SolA15), eFlour 660, eBioscience), antibody-conjugate was applied and then left overnight, followed by washes and mounting with DAPI medium. The next morning secondary antibody, Rabbit anti-mouse Alexa Flour 594 (Fisher) for TRAP or Goat anti-mouse Alexa Flour 594 (Fisher) for BSP was applied and incubated at room temperature for 2 hours. Next, EverBrite Hardset Mounting Medium with DAPI (Biotium) was applied and coverslips placed. The slides were scanned via Zeiss Axio Scan Z.1 (Zeiss). For cell counting on immunofluorescence images, 2 independent blind reviewers were employed to count cells in cortical bone. For counting cells in bone marrow, single fluorescent channel images were imported into Image J. The color image was split into three separate channels and then appropriately thresholded. Automatic counting was achieved through “Analyze particles” in Image J software.

### Statistical analysis

The microCT data was analyzed by two-way ANOVA to examine the effects of the ovariectomy over time on both mouse strains as well as differences in mean values between the mice. The number of stem cells were also analyzed via a two-way ANOVA. Cell counting from immunofluorescence studies were compared via Mann Whitney t-test. All other data were compared via a one-way ANOVA. p<0.05 was considered significant. Generation of graphs and data analysis was performed on GraphPad Prism 7 (California, USA). Data represented are mean ± SD.

## Results

### p21^-/-^ mice are protected against bone loss post-OVX

**Fig 1:**
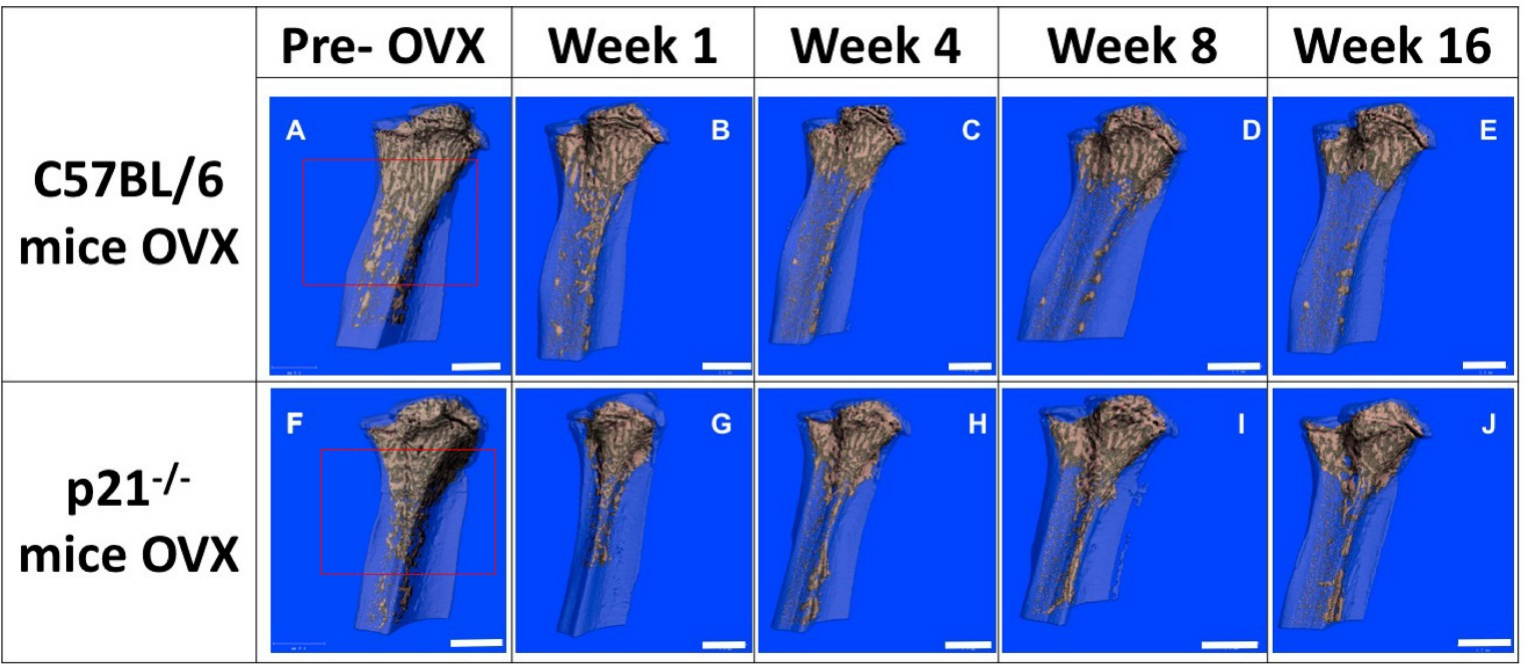
Panel of representative microCT images showing bone morphology between 0 and 16 weeks post-OVX and quantitative analysis of bony parameters. (A-E) C57BL/6 mice (F-J) p21^-/-^ mice. (A and F) pre-OVX (B-E & G-J) post-OVX. (A&F). Scale bar: 1mm.

Representative microCT images of the tibiae of C57BL/6 mice show prominent bone loss post-OVX (Fig 1(A)–1(E), Figure A in S1(A)–S1(D) File, Figure B in S1 File) that continues throughout 16 weeks of repeated measures imaging. Specifically, in C57BL/6 mice, there is a rapid and dramatic reduction of trabecular bone in the metaphyseal region that can be observed as early as 1 week post-OVX surgery (Fig 1(A) and 1(B), Figure A in S1(A) and S1(B) File). Although p21^-/-^ mice also demonstrate noticeable changes in bone morphology post-OVX surgery (Fig 1(F)–1(J), Figure A in S1(E)–S1(H) File) over the 16-week imaging period, the bone loss is not as prominent compared to C57BL/6 mice.

**Fig 2:**
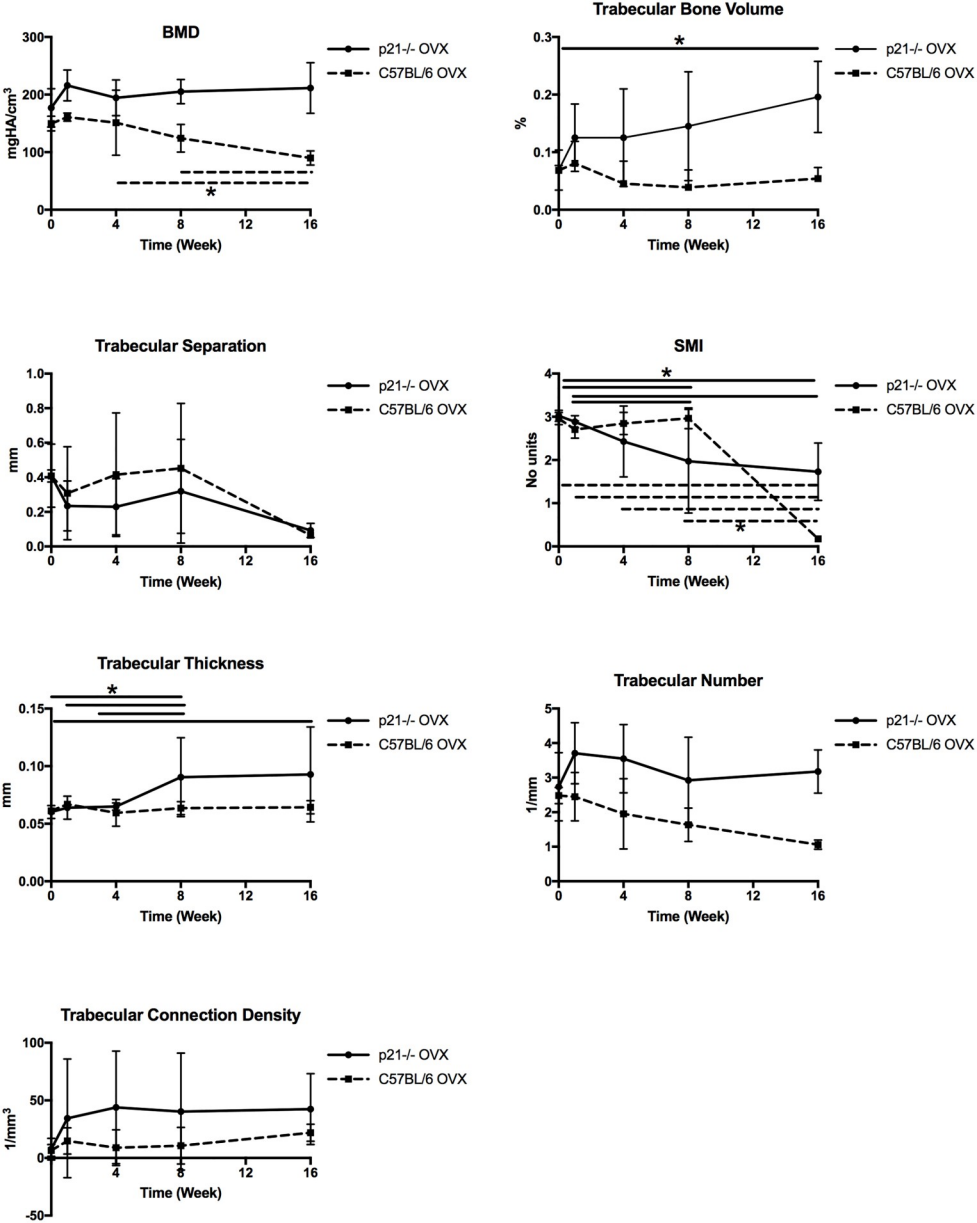
Bone histomorphometry from microCT images of right tibia. p<0.05. HA, hydroxyapatite. Bone mineral density, an important determinant of osteoporosis remains unchanged in p21^-/-^ mice. There are significant differences between both genotypes at W4, W8 and W16. p<0.05. *indicates significant differences within genotypes between time points.

Differences in bony parameters between the mice groups were assessed quantitatively and are presented in the graphs in Fig 2 and Tables A-G in S1 File. The two-way ANOVA shows that there is significant difference between the two strains at all time points and in all parameters. C57BL/6 mice demonstrated significant loss of BMD post-OVX and by 16 weeks, more than 40% of the total BMD was lost compared to pre-OVX animals. p21^-/-^ mice on the other hand, demonstrated no significant changes (pre vs. post-OVX) in BMD over the 16 weeks of the experiment, in fact, a 20% increase in mean BMD was observed. Trabecular bone volume showed a significant increase in the p21^-/-^ mice between baseline and 16-week time point (p<0.015). The structure model index signifies the plate or rod like geometry of trabecular bone structures where SMI = 0 indicates plate-like, 3 indicates rods and 4 indicates solid spheres. Both p21^-/-^ and C57BL/6 mice have an SMI~3 pre-OVX and both show a decline in SMI. At the 16-week time point, p21^-/-^ mice show an SMI close to 1.72 and C57BL/6 has an SMI close to 0.17, implying plate like trabecular structures in both mice.

### Mesenchymal stem cells (MSCs)

To determine potential mechanism behind the observation that p21^-/-^ mice were protected from OXV induced bone loss, the number of undifferentiated mesenchymal stem cells in the bone marrow were enumerated via flow cytometry (Figure C in S1 File). It was found that by 8 weeks post-OVX there was no significant difference in the number of MSCs between both strains of mice, however, by 16 weeks post-OVX, C57BL/6 mice presented with significantly increased numbers of undifferentiated MSCs compared to p21^-/-^ mice, yet the overall number of MSCs was lower in both mice strains compared to 8 weeks post-OVX.

### Osteoclasts and osteoblasts

**Fig 3:**
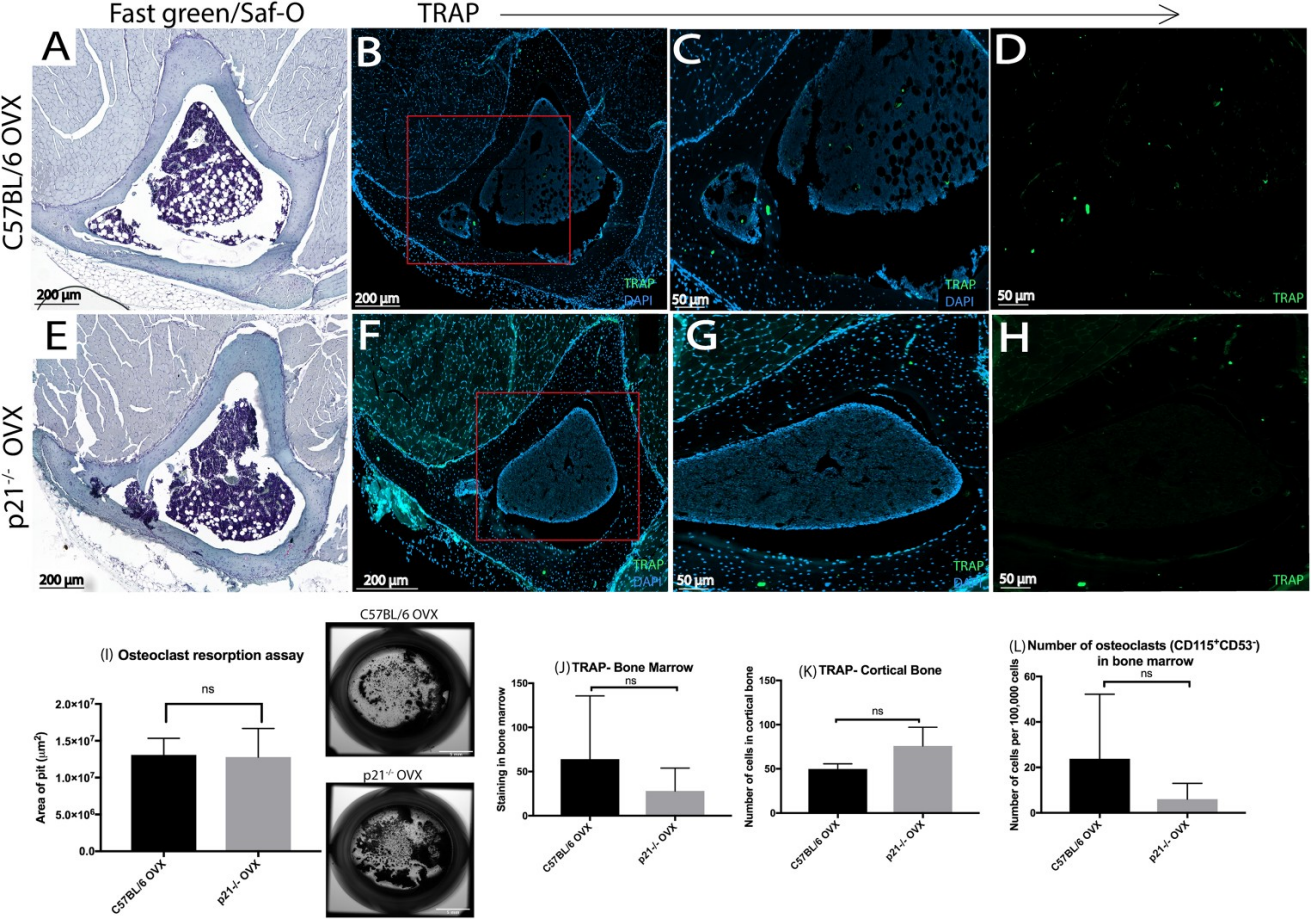
TRAP staining 8 weeks post-OVX (A&E) Fast green and SafO staining of (A) C57BL/6 and (E) p21^-/-^ mice (B-H) DAPI/TRAP staining. (C-D and G-H) are magnified images of (B& F) respectively. (I) Osteoclast resorption assay illustrating pit formation by osteoclasts in C57BL/6 and p21^-/-^ mice post-OVX. Both mice groups show similar area of pit formation. Enumeration of TRAP^+^ cells in (J) bone marrow and (K) cortical bone (L) Number of osteoclasts in the bone marrow measured via flow cytometry.

OVX is known to skew the homeostatic balance in bone in favor of resorption. Therefore, the number and functionality of osteoclasts was investigated (Fig 3(A)–3(L), Figure D in S1 File). No obvious differences in TRAP staining intensity or localization was observed between p21^-/-^ vs. C57BL/6 mice (Fig 3(A)–3(H)). To confirm this finding, the number of TRAP^+^ cells were enumerated, and there were no significant differences between the two strains in the bone marrow or the cortical bone (Fig 3(J)–3(K)). To further strengthen this result, the number of osteoclasts (CD115^+^CD53^-^) within the bone marrow of each mouse strain was enumerated via flow cytometry, and no significant differences between the strains were observed (Fig 3L). Finally, the functionality of the osteoclasts were also assessed via bone resorption assay (Fig 3I), with no significant differences being observed.

**Fig 4:**
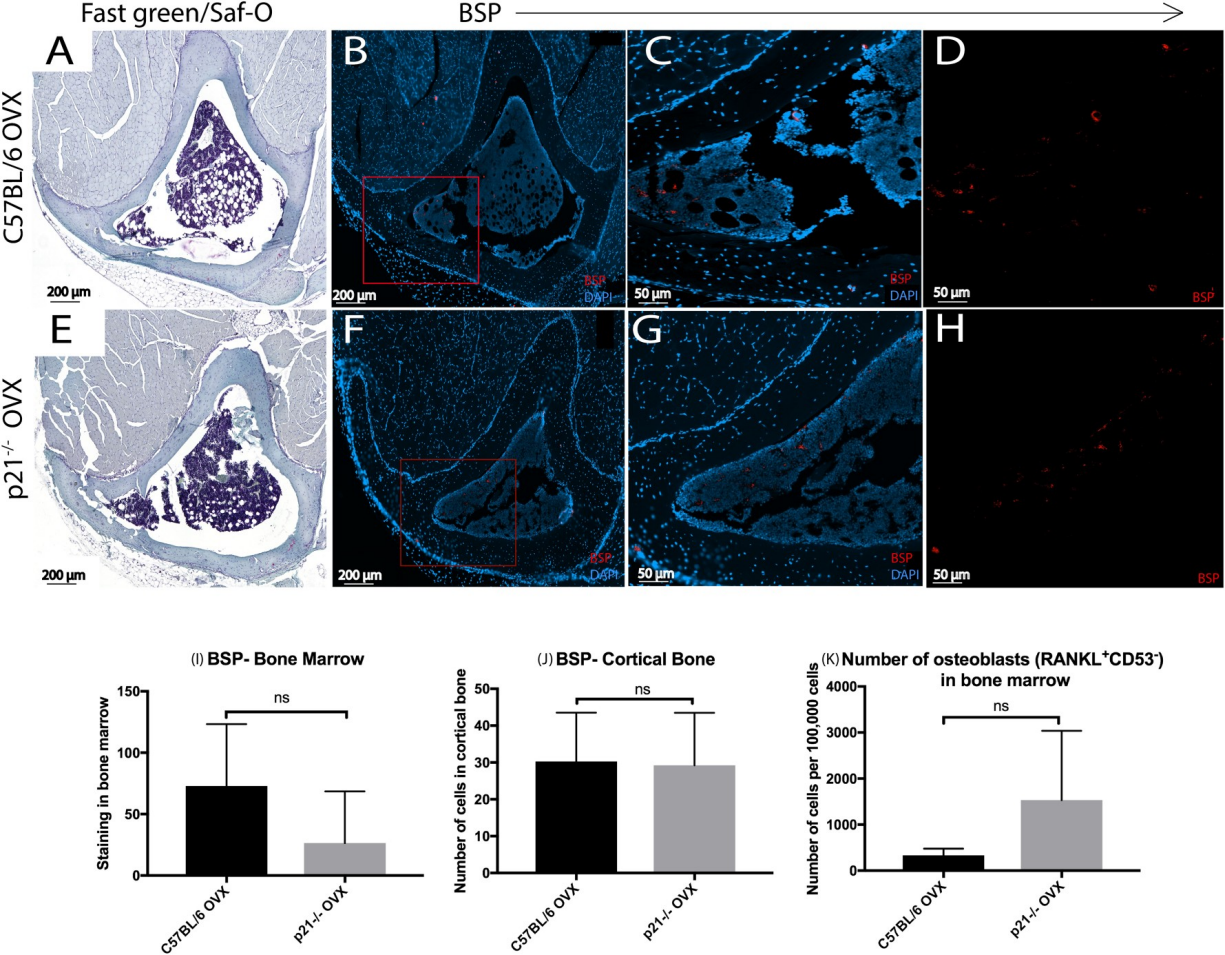
BSP staining 8 weeks post-OVX. (A) C57BL/6 (B) p21^-/-^ fast green/saf-O staining for histology. (B-H) DAPI/BSP staining. (C-D and G-H) are magnified images of (B& F) respectively. Enumeration of BSP^+^ cells in (I) bone marrow and (J) cortical bone (K) Number of osteoblasts in the bone marrow measured via flow cytometry.

Bone sialoprotein (BSP), a marker for mature osteoblasts was employed to determine if there were any differences in the localization of osteoblasts in the trabecular and cortical bone of the tibiae in C57BL/6 vs. p21^-/-^ mice post-OVX (Fig 4, Figure E in S1 File). BSP^+^ staining of similar intensity and localization was observed in both mouse stains post-OVX at 8 weeks. Similar to what was observed with TRAP staining, BSP staining was similar between mice strains by 8 weeks in the bone marrow and cortical bone (Fig 4(J)–4(K)). Enumeration of osteoblasts from a bone marrow aspiration via flow cytometry confirmed these findings, as significant differences between mice was observed (Fig 4K).

### Cell proliferation

**Fig 5:**
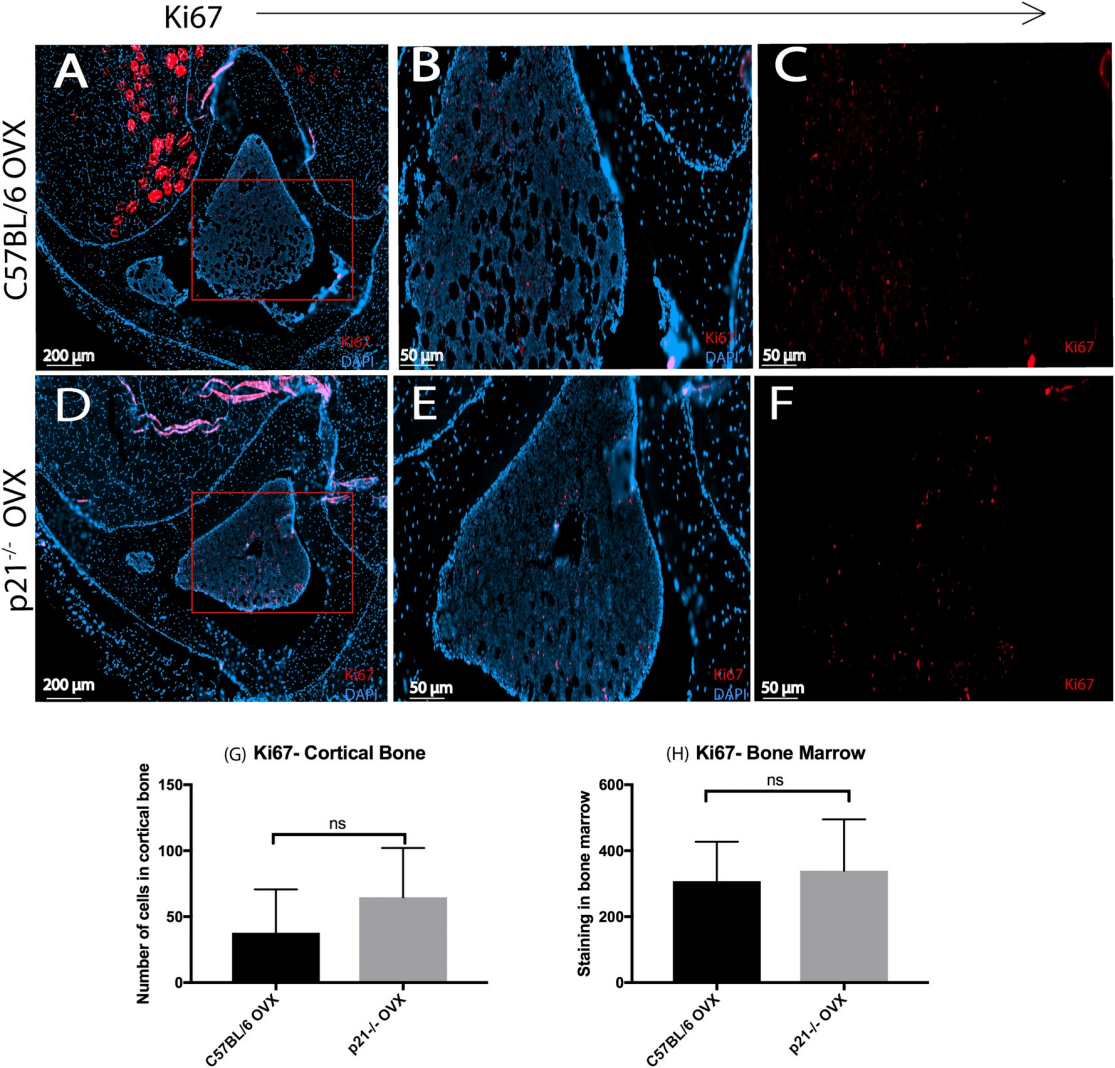
Ki67 staining 8 weeks post-OVX. (A-F) DAPI/Ki67 staining. (B-C and E-F) are magnified images of (A& D) respectively. Enumeration of Ki67^+^ cells in (G) cortical bone and (H) bone marrow.

Since p21 plays a critical role in the regulation of the cell cycle, we stained bone sections of both mice strains for actively dividing cells using the Ki67 marker (Fig 5 and Figure F in S1 File). Both mice show similar staining in the cortical bone and bone marrow.

## Discussion

p21^cip1/waf1^ is regulated by the tumor suppressor gene p53 and plays an important role in cell cycle regulation. Inhibition of p21 has been implicated in regeneration of tissues such as liver [16], and more recently bone regeneration after fracture [12]. The exact mechanism behind p21 regulated regeneration remains elusive despite studies suggesting that enhanced proliferation of cells may be a contributing factor. The results from the current study demonstrate that p21^-/-^ mice are protected against bone loss post-OVX, while C57BL/6 mice show significant bone loss in spite of similar cell numbers in the bone marrow (MSCs, osteoblasts and osteoclasts). Since inhibition of p21 in chondrocytes induces their apoptosis and therefore could influence skeletal development [11], it is possible that differences in endochondral ossification between these strains may contribute to the findings. However, when the thickness of growth plate was assessed, similar to a study by Gerber et al. [17], no significant differences between the two mice strains was observed (Figure G in S1 File). These results suggest that the mechanism by which p21^-/-^ mice retain/regenerate bone post-OVX is not likely based on osteoclast vs. osteoblast function, localization and/or number, and therefore may be a result of a ‘short circuit’ in the OVX disease mechanism itself. While the exact cellular mechanism by which estrogen controls metabolic homeostasis and bone resorption is not fully known, it is well established that diminished estrogen levels through OVX surgery has a dramatic and immediate effect on bone homeostasis in mice through which is mediated through an increase in osteoclast numbers and activity [18]. Interestingly, a direct link has been demonstrated between p21 and estrogen regulation, with previous studies showing that estrogen is a potent inducer of p21 expression [19]. Furthermore, p21 can regulate the expression of the estrogen receptor [20], and can therefore act as a potent inhibitor of the estrogen signaling cascade [21]. Hypothetically then, if the pathway by which estrogen regulates bone homeostasis is interrupted by OVX (e.g. decreased estrogen levels) and p21 is a negative regulator of estrogen signaling, then a double knock-down/knockout of estrogen and p21 respectively, may result in the expected bone loss phenotype being absent. While this hypothesis has yet to be tested, it is supported by our previous study of the role of p21 in bone regeneration, since p21^-/-^ mice demonstrated enhanced bone regeneration after a burr-hole injury compared to C57BL/6 mice, yet no difference in osteoblast vs. osteoclast function was observed [12]. If p21 is downstream and inhibits estrogen signaling, then it may be possible for a p21^-/-^ mice on an estrogen competent background to show the opposite effect vs. when estrogen is removed (e.g. favoring bone formation vs. resorption).

## Conclusions

Our findings demonstrate that p21^-/-^ mice are protected against osteopenia post-OVX compared to C57BL/6 mice. These results suggest a larger role of p21 in bone homeostasis and potentially points to a direct relationship between p21 and estrogen. A better understanding of the mechanism behind estrogen and p21 related bone formation may result in novel therapies for bone repair and/or osteoporosis.

## Supporting information

S1 File Figure A: **3D slice of tibia focused on proximal tibia**. (A-D) C57BL/6 mice and (E-H) p21^-/-^ mice. (A&E) Week 0 (B&F) Week 4 (C&G) Week 8 (D&H) Week 16. Scale bar: 1mm. Figure B: **Saf-O/Fast green staining of 4 week tibiae**. (A) C57BL/6 (B) p21^-/-^ mice. C57BL/6 mice already show significant reduction in trabecular bone compared to p21^-/-^ mice. Figure C: **Flow cytometry data indicating number of cells in bone marrow** (A) Sca1+ and CD140a+ undifferentiated mesenchymal stem cells at 8 weeks and 16 weeks post OVX. A two-way ANOVA shows significance between time points and between strains at 16-week time point. Figure D: **Slices stained with DAPI and secondary antibody but not primary antibody (TRAP)** to control for auto-fluorescence. Figure E: **Slices stained with DAPI and secondary antibody but not primary antibody (BSP)** to control for auto-fluorescence. Figure F: **Slices stained with DAPI Ki67-conjugate antibody** to control for auto-fluorescence. Figure G: **There are no significant differences between the thickness of the epiphyseal cartilage** thickness of C57BL/6 and p21^-/-^ mice. p<0.05. Table A: BMD. Table B: Trabecular Connectivity Density. Table C: SMI. Table D: Trabecular Bone Volume. Table E: Trabecular Number. Table F: Trabecular Separation. Table G: Trabecular Thickness.
(DOCX)

S1 Checklist NC3Rs ARRIVE Guidelines Checklist (fillable).pdf. (PDF)
