# Supplementary material for: Absence of p21(WAF1/CIP1/SDI1) protects against osteopenia and minimizes bone loss after ovariectomy in a mouse model
Source: PLoS One. 2019 Apr 10;14(4):e0215018. doi: 10.1371/journal.pone.0215018 (PMC6457871; doi:10.1371/journal.pone.0215018)
Supplement: S1 File — Figure A: 3D slice of tibia focused on proximal tibia. (A-D) C57BL/6 mice and (E-H) p21-/- mice. (A&E) Week 0 (B&F) Week 4 (C&G) Week 8 (D&H) Week 16. Scale bar: 1mm. Figure B: Saf-O/Fast green staining of 4 week tibiae. (A) C57BL/6 (B) p21-/- mice. C57BL/6 mice already show significant reduction in trabecular bone compared to p21-/- mice. Figure C: Flow cytometry data indicating number of cells in bone marrow (A) Sca1+ and CD140a+ undifferentiated mesenchymal stem cells at 8 weeks and 16 weeks post OVX. A two-way ANOVA shows significance between time points and between strains at 16-week time point. Figure D: Slices stained with DAPI and secondary antibody but not primary antibody (TRAP) to control for auto-fluorescence. Figure E: Slices stained with DAPI and secondary antibody but not primary antibody (BSP) to control for auto-fluorescence. Figure F: Slices stained with DAPI Ki67-conjugate antibody to control for auto-fluorescence. Figure G: There are no significant differences between the thickness of the epiphyseal cartilage thickness of C57BL/6 and p21-/- mice. p<0.05. Table A: BMD. Table B: Trabecular Connectivity Density. Table C: SMI. Table D: Trabecular Bone Volume. Table E: Trabecular Number. Table F: Trabecular Separation. Table G: Trabecular Thickness. (DOCX) [file pone.0215018.s001.docx]

**S1. Supplementary Information**

**
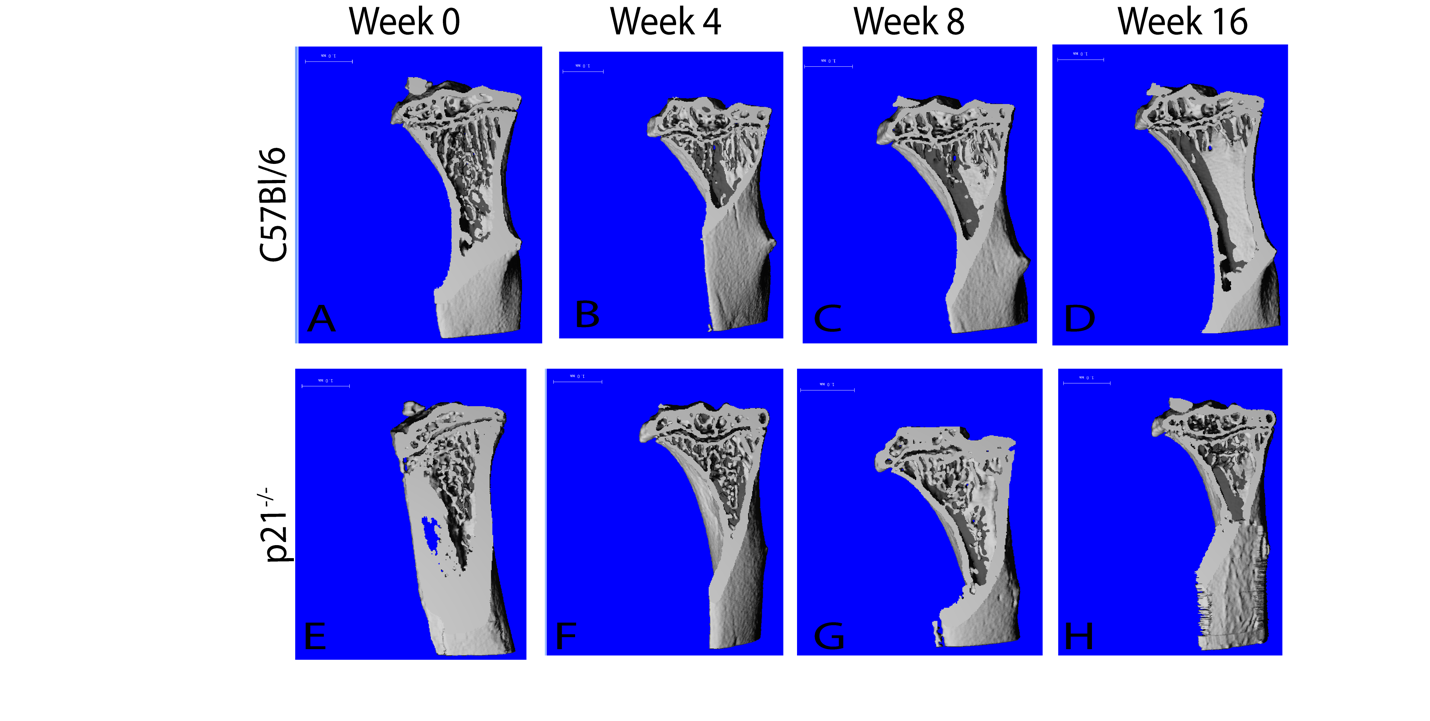
**

**Figure A: 3D slice of tibia focused on proximal tibia.** (A-D) C57BL/6 mice and (E-H) p21^-/-^ mice. (A&E) Week 0 (B&F) Week 4 (C&G) Week 8 (D&H) Week 16. Scale bar: 1mm.


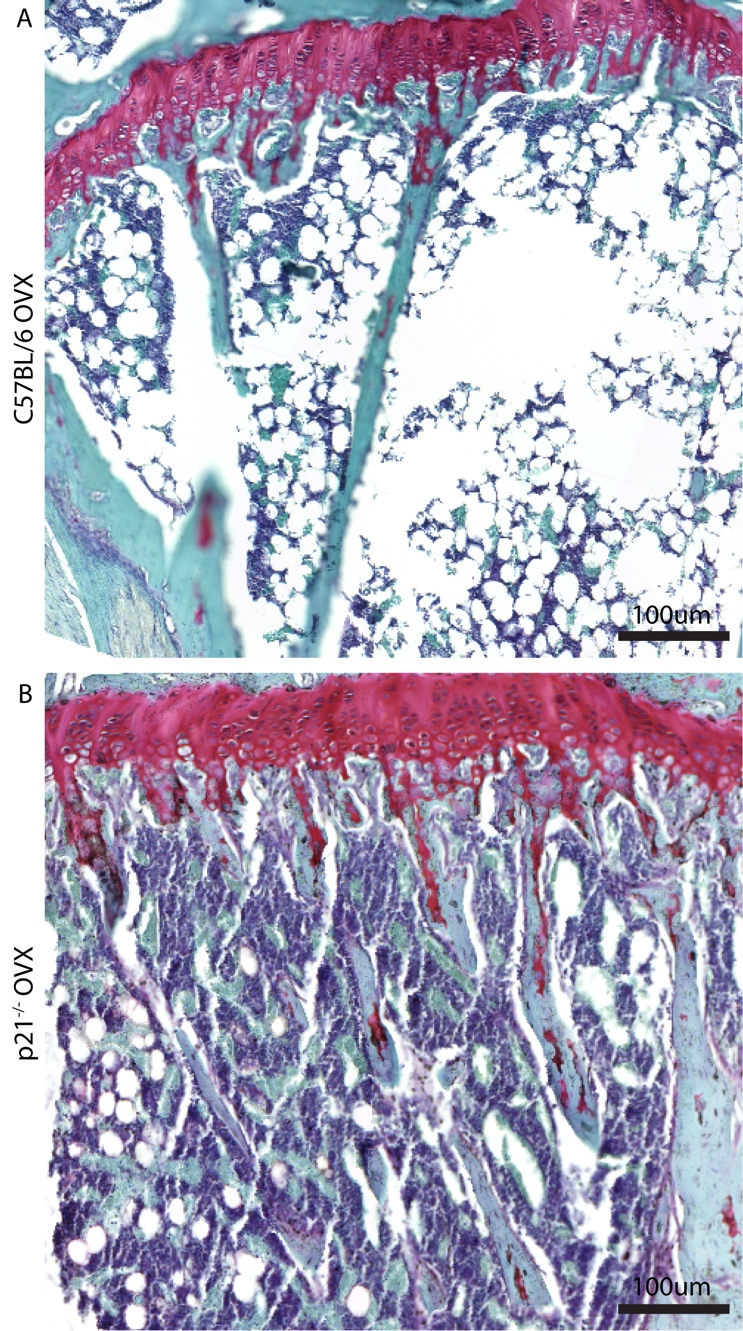


Figure B : **Saf-O/Fast green staining of 4 week tibiae**. (A) C57BL/6 (B) p21^-/-^ mice. C57BL/6 mice already show significant reduction in trabecular bone compared to p21^-/-^ mice.


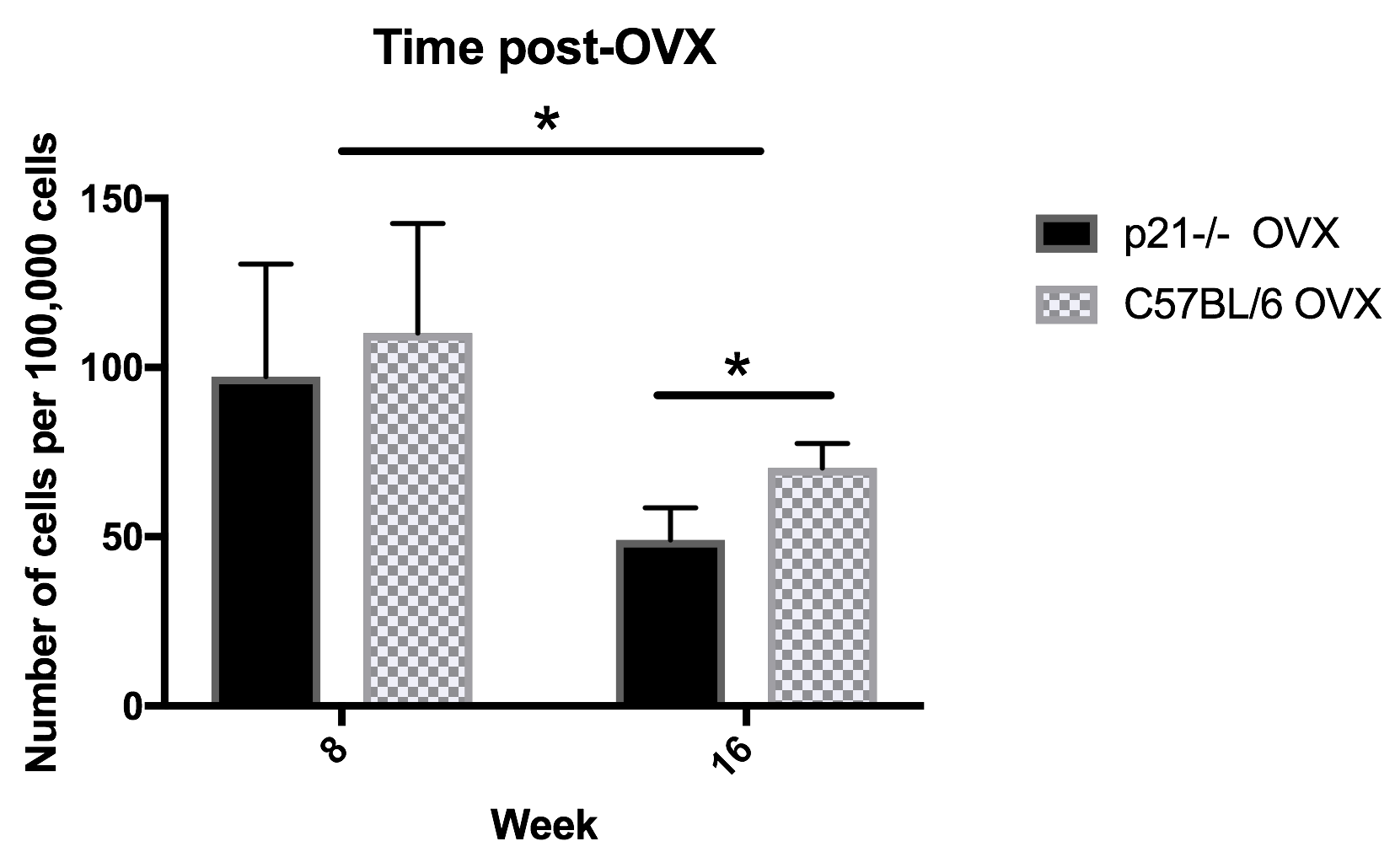


Figure C: **Flow cytometry data indicating number of cells in bone marrow** (A) Sca1+ and CD140a+ undifferentiated mesenchymal stem cells at 8 weeks and 16 weeks post OVX. A two-way ANOVA shows significance between time points and between strains at 16-week time point.


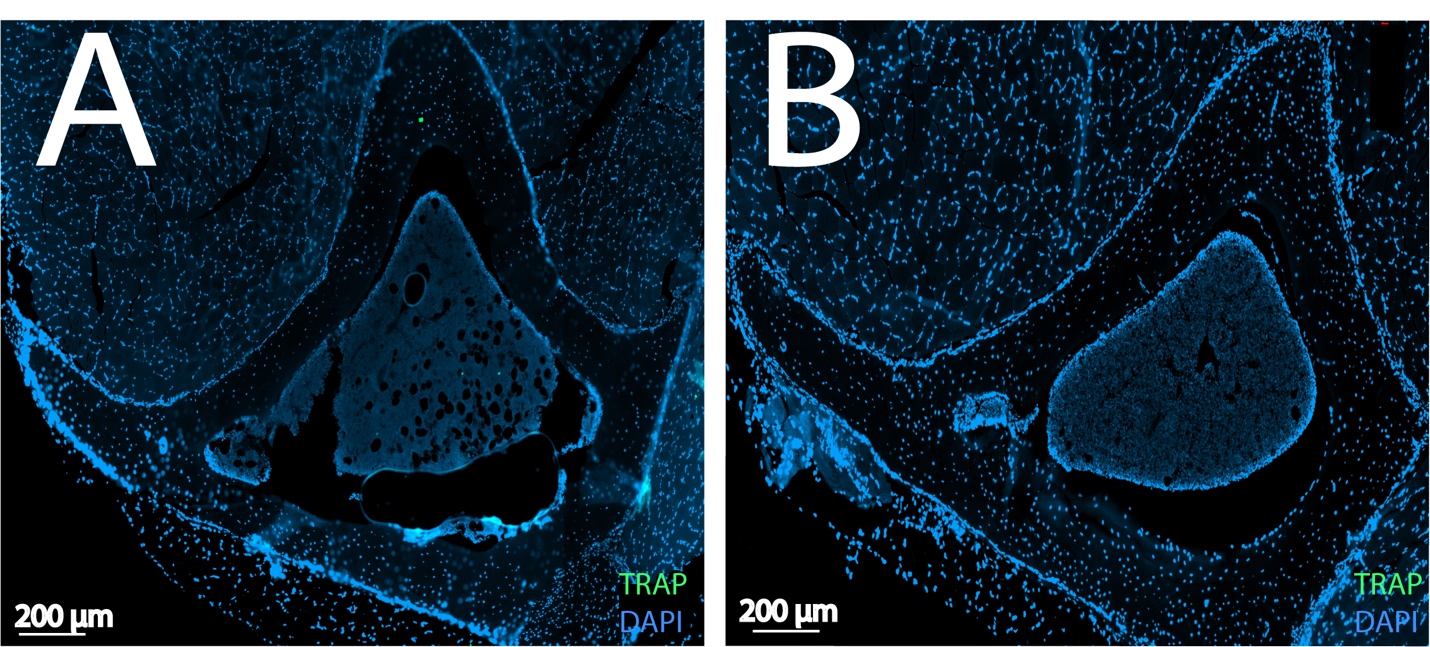


Figure D: **Slices stained with DAPI and secondary antibody but not primary antibody** **(TRAP)** to control for auto-fluorescence.


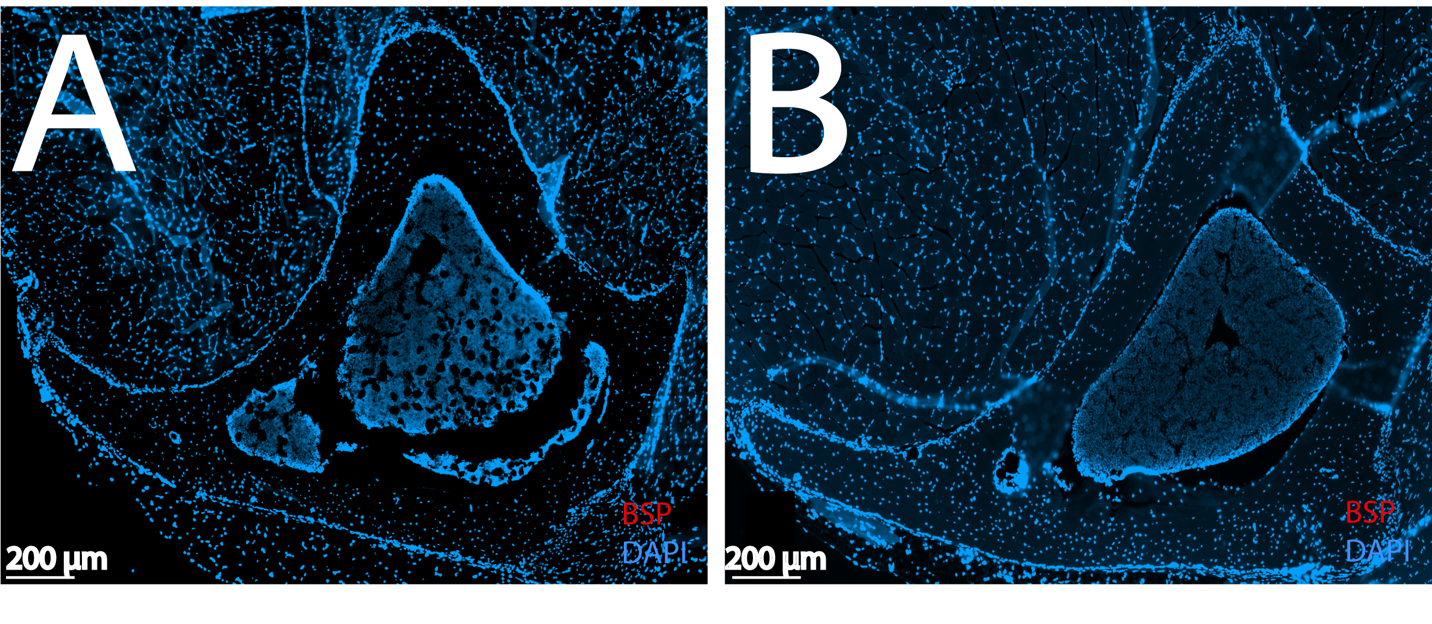


Figure E: **Slices stained with DAPI and secondary antibody but not primary antibody (BSP)** to control for auto-fluorescence.


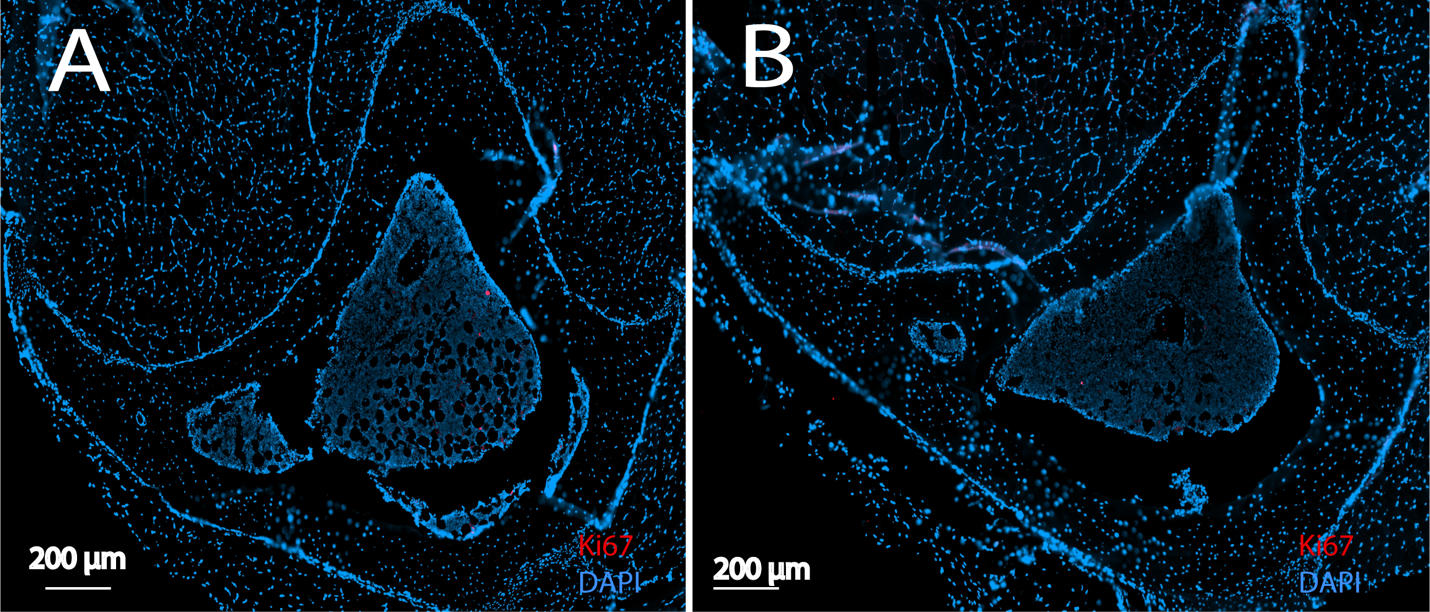


Figure F: **Slices stained with DAPI Ki67-conjugate antibody** to control for auto-fluorescence


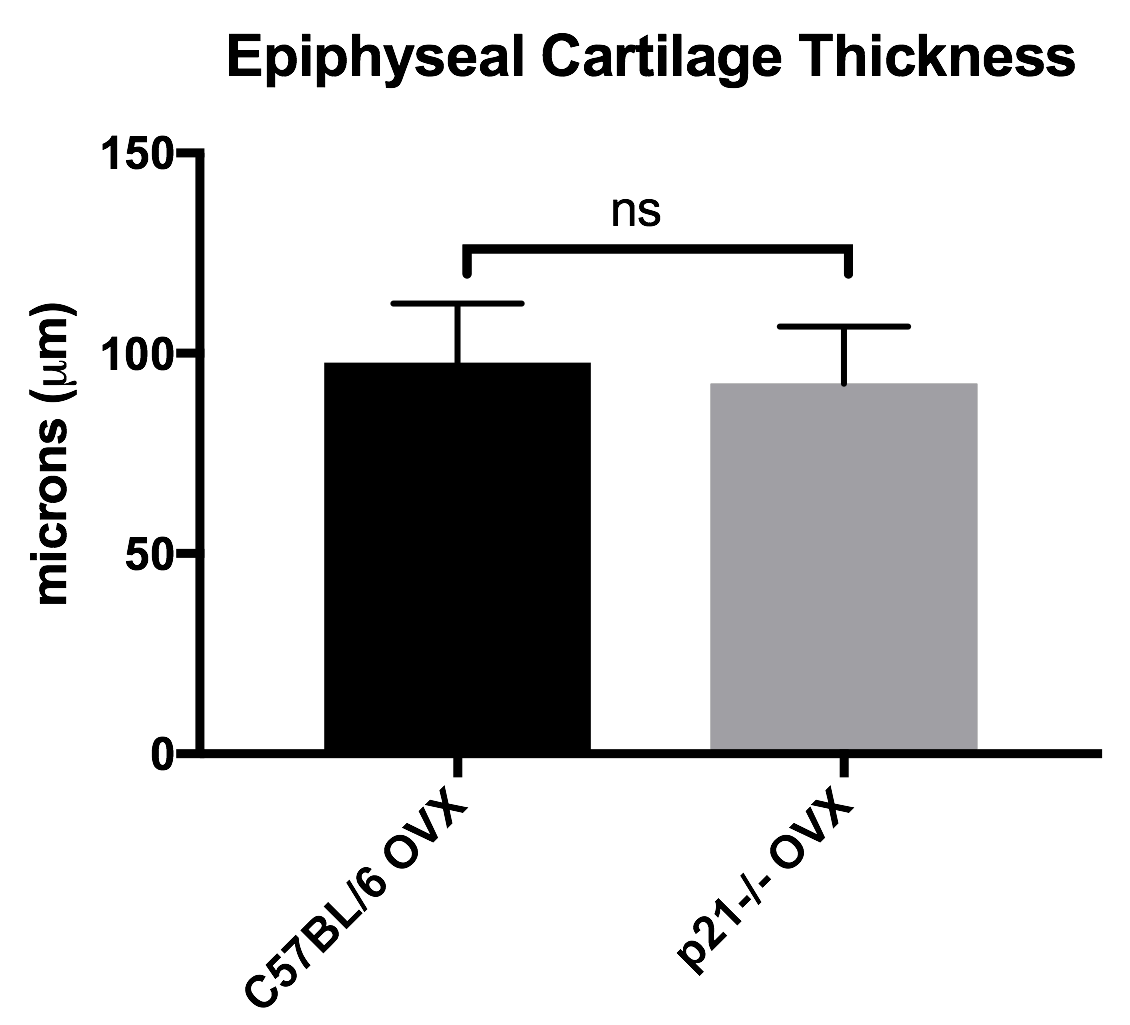


Figure G: **There are no significant differences between the thickness of the epiphyseal cartilage** thickness of C57BL/6 and p21^-/-^ mice. p<0.05.

Table A: BMD

| **Time (Week)** | **p21-/- OVX** | **C57BL/6 OVX** |
| --- | --- | --- |
| 0 | 176.9728167 | 149.64528 |
| 1 | 215.7961 | 160.8668143 |
| 4 | 194.3537143 | 151.2284875 |
| 8 | 205.0056333 | 124.15725 |
| 16 | 211.3499333 | 89.96956667 |

Table B: Trabecular Connectivity Density

| **Time (Week)** | **p21-/- OVX** | **C57BL/6 OVX** |
| --- | --- | --- |
| 0 | 7.69405 | 6.5944 |
| 1 | 34.40861111 | 14.80951429 |
| 4 | 43.92692857 | 8.9866625 |
| 8 | 40.30638333 | 10.707375 |
| 16 | 42.4707 | 21.94086667 |

Table C: SMI

| **Time (Week)** | **p21-/- OVX** | **C57BL/6 OVX** |
| --- | --- | --- |
| 0 | 3.020066667 | 2.95988 |
| 1 | 2.8863 | 2.70565 |
| 4 | 2.427625 | 2.8451 |
| 8 | 1.971466667 | 2.96466 |
| 16 | 1.7281 | 0.1752 |

Table D: Trabecular Bone Volume

| **Time (Week)** | **p21-/- OVX** | **C57BL/6 OVX** |
| --- | --- | --- |
| 0 | 0.0688 | 0.06826 |
| 1 | 0.125155556 | 0.0806 |
| 4 | 0.125242857 | 0.0454 |
| 8 | 0.145033333 | 0.0389875 |
| 16 | 0.195833333 | 0.054233333 |

Table E: Trabecular Number

| **Time (Week)** | **p21-/- OVX** | **C57BL/6 OVX** |
| --- | --- | --- |
| 0 | 2.736233333 | 2.48022 |
| 1 | 3.705233333 | 2.446442857 |
| 4 | 3.546985714 | 1.9521625 |
| 8 | 2.92475 | 1.6351875 |
| 16 | 3.178433333 | 1.0619 |

Table F: Trabecular Separation

| **Time (Week)** | **p21-/- OVX** | **C57BL/6 OVX** |
| --- | --- | --- |
| 0 | 0.409666667 | 0.40826 |
| 1 | 0.234711111 | 0.307985714 |
| 4 | 0.229757143 | 0.4151 |
| 8 | 0.319933333 | 0.4517875 |
| 16 | 0.0928 | 0.064333333 |

Table G: Trabecular Thickness

| **Time (Week)** | **p21-/- OVX** | **C57BL/6 OVX** |
| --- | --- | --- |
| 0 | 0.06015 | 0.06116 |
| 1 | 0.063888889 | 0.066985714 |
| 4 | 0.064957143 | 0.0594375 |
| 8 | 0.090383333 | 0.0636125 |
| 16 | 0.0928 | 0.064333333 |
